# Mapping hypoendemic, seasonal malaria in rural Bandarban, Bangladesh: a prospective surveillance

**DOI:** 10.1186/1475-2875-10-124

**Published:** 2011-05-14

**Authors:** Wasif A Khan, David A Sack, Sabeena Ahmed, Chai Shawi Prue, Mohammad Shafiul Alam, Rashidul Haque, Jacob Khyang, Malathi Ram, Jasmin Akter, Myaing Myaing Nyunt, Douglas Norris, Gregory Glass, Timothy Shields, Md Zahirul Haq, Alejandro Cravioto, David J Sullivan

**Affiliations:** 1 ICDDR, B: International Centre for Diarrhoeal Disease Research, Clinical Sciences Division; 2 Johns Hopkins Malaria Research Institute, JHBSPH, Baltimore, MD, USA

## Abstract

**Background:**

Until recently the Chittagong Hill tracts have been hyperendemic for malaria. A past cross-sectional RDT based survey in 2007 recorded rates of approximately 15%. This study was designed to understand the present epidemiology of malaria in this region, to monitor and facilitate the uptake of malaria intervention activities of the national malaria programme and to serve as an area for developing new and innovative control strategies for malaria.

**Methods:**

This research field area was established in two rural unions of Bandarban District of Bangladesh north of Bandarban city, which are known to be endemic for malaria due to *Plasmodium falciparum*. The project included the following elements: a) a demographic surveillance system including an initial census with updates every four months, b) periodic surveys of knowledge attitude and practice, c) a geographic information system, d) weekly active and continuous passive surveillance for malaria infections using smears, rapid tests and PCR, f) monthly mosquito surveillance, and e) daily weather measures. The programme included both traditional and molecular methods for detecting malaria as well as lab methods for speciating mosquitoes and detecting mosquitoes infected with sporozoites.

**Results:**

The demographic surveillance enumerated and mapped 20,563 people, 75% of which were tribal non-Bengali. The monthly mosquito surveys identified 22 *Anopheles *species, eight of which were positive by circumsporozoite ELISA. The annual rate of malaria was close to 1% with 85% of cases in the rainy months of May-October. Definitive clustering identified in the low transmission season persisted during the high transmission season.

**Conclusion:**

This demographically and geographically defined area, near to the Myanmar border, which is also hypoendemic for malaria, will be useful for future studies of the epidemiology of malaria and for evaluation of strategies for malaria control including new drugs and vaccines.

## Background

Bangladesh is one of the 109 countries listed by the World Health Organization as having endemic malaria. Within Bangladesh, 13 of the 64 districts are considered to have endemic malaria, with 26.9 million people living in these 13 districts, a population larger than many African countries, which illustrates the scope of the risk from malaria. The government of Bangladesh, with financial assistance from the Global Fund for AIDS, TB and Malaria, recently initiated programmes to control malaria in these districts. The primary interventions for the programme have focused on distribution of insecticide-treated bed nets, malaria diagnosis at the field level using rapid diagnostic tests (RDT), and artemisinin-based combination therapy (ACT) as the first-line of treatment. Reports from district hospitals in these endemic areas demonstrate a reduction in the numbers of cases detected since the programme started in 2008; however, it is not clear if this reduction is due to the programme activities, to climate related variability in rates from year to year, or differences in the ascertainment of cases. These reports are based on cases treated at health facilities, which may not reflect the true rates of malaria since many patients may not seek treatment or reach a facility. Thus, the true rate of malaria in the endemic districts is not well documented.

A cross-sectional survey for malaria infection was conducted in 2007 by International Centre for Diarrhoeal Disease Research, Bangladesh (ICDDR, B) with BRAC to establish a baseline estimate of malaria prevalence in the population living in the 13 malaria endemic districts [1]. This cross-sectional survey showed a malaria prevalence of 13% by RDT in the Chittagong Hill Tracts (15.5, 10.7 and 6.8 percent in Khagrachari, Bandarban and Rangamati districts respectively) with the overall prevalence of 45 per thousand population of fever associated with malaria in these three Hill districts. About 89% of the infections were caused by *Plasmodium falciparum*, 5% by *Plasmodium vivax*, and the remaining by mixed infection. Asymptomatic prevalence in five southeastern districts was 40/1,000 versus 2/1,000 population in the eight northeastern districts. Such high rates of asymptomatic malaria infection suggested a need for further surveillance and control measures.

While most control strategies, as well as surveillance methods, are focused on symptomatic malaria, transmission of malaria occurs because of circulating gametocytes often from asymptomatic individuals. Unfortunately, little is known about the epidemiology of gametocyte infection in Bangladesh, but reducing transmission will depend on an improved understanding of all phases of malaria infection in both humans and the insect vectors.

Though the malaria burden among symptomatic patients is beginning to be understood for Bangladesh, there may be specific subgroups, which are especially vulnerable to increased mortality and morbidity from malaria, especially pregnant women. In other geographic areas, malaria increases the risk of mortality for the mother during pregnancy and increases the risk for low birth weight in the infant [2-4].

In order to better define the epidemiology of malaria in an endemic area of Bangladesh, the ICDDR,B in collaboration with the Johns Hopkins Malaria Research Institute (JHMRI) established a surveillance system for malaria in an area of the Chittagong Hill Tracts in Southeast Bangladesh. The area selected for the surveillance was the two unions north of Bandarban town (Kuhalong and Rajbila), each with a population of about 10,000 people. The selection of these unions was based on data from the initial survey showing relatively high rates of malaria due to *P. falciparum*, and their relative accessibility. Residents of these unions are predominantly tribal people who speak their own language and not Bengali. The study area is close to the border with Myanmar and thus provides information of relevance to the Southeast Asia "ecozone" for malaria. This ecozone has been the source for isolates of malaria, which are resistant to multiple drugs. Thus, the Bandarban study area was felt to be an appropriate site for monitoring for emergence of drug resistance in the future. Being hypoendemic, it seemed that the study site could be a potential site for future studies of innovative control measures. Such studies carried out in the Bandarban field area of Bangladesh may be relevant to other countries in the Southeast Asia ecozone and potentially to other areas which are becoming hypoendemic as control measures reduce rates of malaria in previously hyperendemic areas.

Although the two unions selected for the surveillance activity are adjacent, they also have some contrasting features. Kuhalong is more forested and hilly, whereas Rajbila tends to have more rice fields. The earlier baseline survey conducted in a small number of subjects suggested that Kuhalong had higher rates of malaria (28% prevalence versus 4% for Rajbila); however, based on these small numbers it was not possible to establish with certainty which union had a higher disease burden [1]. Kuhalong is closer to Bandarban town, which has a hospital, while Rajbila is more distant, requiring between one to two hours to drive to the hospital.

The objectives for the project are shown in Table 1. This paper focuses on the methodology and activities used to accomplish these objectives. Subsequent papers will provide specific findings from ongoing studies. This malaria surveillance project intends to complement the national malaria programme as well as to provide a resource for conducting specific studies on malaria.

**Table 1:** Objectives for the Project on Mapping Malaria in Bangladesh

1.	Define the rates of symptomatic and asymptomatic malaria in the study area.
2.	Define risk factors for malaria (symptomatic asymptomatic infection) using both active and passive surveillance.

3.	Establish a demographic surveillance system including a geographic information system as resources for future malaria epidemiologic studies

4.	Understand the changing knowledge attitude and practices regarding malaria, its prevention and treatment among persons in the study area and among health care providers.

5.	Validate diagnostic methods (microscopic examination and rapid diagnostic tests) with PCR as the gold standard.

6.	Understand the epidemiological patterns of gametocyte prevalence as this relates to transmission of malaria.

7.	Define the vectors which transmit malaria in this area.

## Methods

### Approaching the community, the ministry, and stakeholders

When the two unions were identified as areas which seemed to be ideal for the project, the Upazila (an Upazila is the name given for a sub-district in Bangladesh) Health and Family Planning (UHFP) office in the Bandarban district office was contacted to discuss the purpose of the investigation. Since malaria had already been identified as one of the high priority problems in this area, the officer was very cooperative in assisting with the project. UHFP had information about the population in the two unions; however, this information was several years old; thus, the need to carry out new data collection of the population was discussed. The UHFP office provided two experienced staff members to assist in planning the project. These staff members from the UHFP office were very familiar with the unions of Kuhalong and Rajbila, were from the same tribal communities, and were fluent in the tribal languages.

Together with the UHFP staff, discussions were held with local community leaders, including all levels of leaders called Karbari, Headman, Union Parishad Chairman, and the Upazila Vice Chairman, to explain the purpose of the project and the proposed activities, including establishing a demographic surveillance system. Prior to actually starting the project, a meeting was held at the Bandarban Hill District Council Auditorium where most of the District & Upazila level Health & Family Planning Officers, NGO leaders, Upazila Chairman, Union Parishad Chairman & Members, Headmen and Karbaris participated. During the meeting, the project was described, and the participants had the opportunity to ask questions to the investigators. The general response was favorable since malaria was already known to be the major health problem of the area, and because the ICDDR,B has an excellent reputation in the country for its humanitarian services. The leaders were pleased that this effort was being made to help control malaria for their people.

### Baseline population and geographic information

Baseline information on the populations of Kuhalong and Rajbila was collected during March and April, 2009 using locally hired surveillance workers. These workers recorded the numbers of persons as well as the global positioning system (GPS) coordinates of each household. The data was validated for consistency and completeness at the end of each day and problems were resolved the following day by revisiting the households. Random checks were carried out by a senior health assistant to ensure that data were properly noted on the forms.

**Figure 1:**
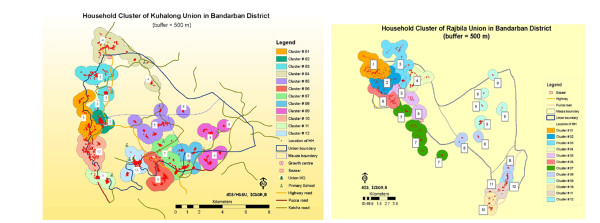
**Map of Kuhalong (A) and Rajbila (B) showing geographic clusters used for surveillance**.

GPS coordinates were imported and mapped in a geographic information system (GIS) using ArcGIS9.2 (ESRI, Redlands, CA). The map demonstrated that households were highly clustered within the unions. The clustering was likely the result of the terrain and hills, which is quite different from the plains of Bangladesh where people are spread more evenly throughout the countryside. These maps were then used to divide the unions into geographic clusters (Figure 1). The union and para (area consisting of several households) numbers were pre-designated by the LGED (Local Government Engineering Department) and these numbers were used in the demographic database.

### Demographic surveillance

Using the baseline listing of the households, teams of surveillance workers visited each house to conduct the census, to administer a detailed survey and to confirm the GPS reading. A unique identifier or respondent ID (RID) was used to identify each person, which included a 1-digit union code, 3-digit cluster code, 3-digit para code, 3-digit household number and a 2-digit individual member number. The format is, therefore, as follows: RID: □-□□□-□□□-□□□-□□. A number was painted on the front door of each house corresponding to the first 10 digits of the RID number to be used by the field workers during subsequent visits. The initial visit included documenting the information on each member of the household, including age, sex, and relationships. Follow-up visits were then conducted every four months to update the census records by collecting vital events, which had occurred during these intervals.

Information on pregnancy was collected by the DSS surveillance during each visit. Thus, the database includes information on pregnancy from a relatively early stage to ascertain pregnancy outcomes as well as any possible complications related to malaria. Pregnancy was documented only by history but not confirmed using pregnancy tests.

The DSS system used certain definitions. A "resident" was a person who resided in the study area continuously for at least three months during the past 12 months. A "visitor" was a person who remained in the study area for at least one week at a time, but stayed in the area less than three months during the past 12 months. The category of "visitor" was needed since the malaria surveillance intended to include all persons present in the surveillance area during the study period regardless of permanence residency. A "visitor" however could become a "resident" during the study if he/she met the defined criteria.

### Knowledge, attitude and practice

Information was collected from each household to determine the socio economic status and to identify the knowledge, attitudes, and practices (KAP) of the families in the area with regard to malaria, malaria treatment, health seeking behaviour, and use of bed nets. The first round of KAP surveys was administered at the same time as the initial DSS survey and subsequent KAP surveys are scheduled to be carried out periodically to observe changes over time.

### Field worker and supervisory staff

The field work was conducted by locally hired surveillance workers (SW). The SWs were hired through a competitive process (written and oral exams), with nearly all being tribal people. Thus, the SWs spoke the local languages and were familiar with the customs and geographical features of the area. Most of them had worked in other non-government organizations and had some experience with data collection.

**Figure 2:**
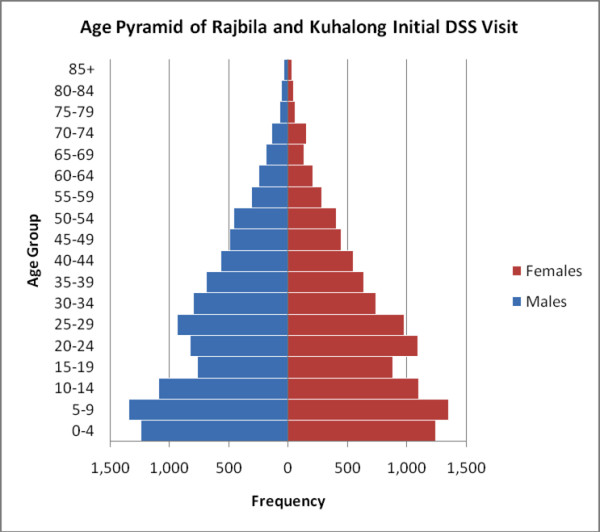
**Population pyramid showing age and sex distribution of the study area**.

The workers were trained in the specific research methods to be undertaken, including approaching and communicating with persons in the community, obtaining informed consent, obtaining the relevant survey information and filling in the forms properly, weighing subjects, obtaining finger prick blood, preparation of blood slides, collection of blood using the rapid diagnostic kits, interpreting the results of the rapid test, obtaining blood on filter paper for PCR, and mosquito trapping procedures. They were also trained in the appropriate use of anti-malarial drugs according to the national guidelines as well as the guidelines for referral of severe cases. Figure 2 illustrates the field work being conducted in this rural, forested area of Bangladesh.

Hiring of SWs was initiated in June 2009 and their field work in Kuhalong began in October 2009. Field work in Rajbila began in April 2010. The SWs in each union were divided into five teams with two persons in each team. One team was designated to carry out the DSS survey, three teams were designated for the active malaria surveillance and one team was designated to conduct mosquito surveillance. The teams were supervised at the first level by a Field Assistant (FA), and at the second level by the Field Research Manager (FRM) and a medical officer (MO). The FA was selected from among the SWs eight months after initiation of the project based on his/her performance in the field. The MO was a local, qualified, physician who had completed post-graduate studies in Public Health from Australia.

### Consent process

Before initiating the field activities, an experienced Field Research Manager (FRM) was transferred from another project to help with communications with the community. Being a tribal person with more than 20 years experience working in field projects with the ICDDRB, he was familiar with the local customs and language as well as with field epidemiologic methods. Together with other project staff, he visited the communities and discussed the project with local leaders and administrative officials. When the teams went to the households to request their participation in the study, they obtained the informed consent of the head of each household or his/her representative. This consent allowed for the collection of information for the Family Visit Register (FVR), which included the names of all the household members, the relationship with the household head, age and sex. A copy of consent form and completed FVR were given to the head of the household.

In addition, for those households or persons who were included in the active and passive surveillance or the mosquito surveillance, written informed consent was obtained from each household or subject (or their legal parent/guardians in case of minor). Copies of the consent documents were given to the individuals who were enrolled.

### Weather data

Weather information was obtained from Bandarban from the Ministry of Agriculture, Soil Resources Development Institute which had been measuring the following parameters daily for over ten years: temperature (maximum, minimum, dry and wet bulb), dew point, humidity, soil temperature at 5, 10, 30 and 50 cm, wind direction and speed, span of sunshine, evaporation and daily rainfall.

### Active surveillance for malaria

Active surveillance was conducted on a random sample of persons throughout the year. Each of the 12 clusters had roughly equal numbers of households. The sampling design was based on clusters stratified into 3 groups (Clusters 1-4 = cluster group A; clusters 5-8 = cluster group B and clusters 9-12 = cluster group C). Sampling was also stratified by age: 6m- < 5y, 5y- < 15y and > 15 yrs. Information and blood samples were obtained every week from four individuals from each age group in each cluster group (4 × 3 = 12 individuals). Two of the 12 subjects were followed for one year as part of a longitudinal surveillance process. These two persons were selected at random from the DSS listing for the union. Using this sampling procedure, a total of 624 samples, equally divided between the three age groups, were collected over the course of one year. Additionally, all pregnant women were invited to participate in both active and longitudinal surveillance.

During visits for the active surveillance, information related to symptoms of malaria were obtained, and a blood sample was obtained for a malaria smear for microscopic examination, a rapid diagnostic test (FalciVax, Zephyr Biomedicals, Verna, Goa, India), and a filter paper sample for PCR. The blood sample was obtained regardless of symptoms. This rapid test identifies antigens for both *P. falciparum *and *P. vivax*. Persons with a positive malaria smear or rapid test were provided with Coartem^®^, according to the national guidelines [5].

### Passive surveillance for malaria

Information from symptomatic cases of malaria was obtained by field workers during their visits to the community. These were detected either directly from family members who alerted a SW about a person with fever or from another group of health workers from a nongovernmental organization (BRAC) which were providing services for malaria as part of the national programme. In either case, when a person with fever became known to an SW, the patient was visited, a blood sample was obtained for a malaria smear, a RDT (FalciVax), and a sample was taken for PCR using filter paper. If the rapid test or the smear was positive, the patient was provided with Coartem, according to the national guidelines[5]. The results of the PCR test was not used to make decisions on treatment since this was considered a research test, and the results were not available in time to make clinical decisions.

Persons with malaria, regardless of whether detected during active or passive surveillance were visited on days 2, 7 and 28 to inquire about the persistence of symptoms and to obtain additional samples for microscopy, rapid test and PCR.

### Mosquito surveillance

For the entomological investigation, five houses were selected to include houses at both high and low altitude from each of the 12 clusters (total of 60 houses). Entomological collection was done using a CDC light trap. Each trap was placed in the house for at least 12 hours (6-7 PM to 6-7 AM) close to persons sleeping under a bed net. Collections were made from the same five houses in each cluster for five nights, one week per month, throughout the year. After each collection, traps were returned to the field laboratory and the mosquitoes were killed. Mosquitoes were then counted, sorted and identified up to species level. *Anopheles *mosquitoes were preserved in a separate vial (1.5 ml) according to house and species, labelled and caped with silica gel and cotton inside before sending to the ICDDR,B Parasitology Laboratory in Dhaka to reconfirm species designations and assess the presence of sporozoites by ELISA.

### Laboratory facilities

Laboratory facilities were available in Bandarban at the field office, at the ICDDR,B in Dhaka and at the Malaria Research Institute at Johns Hopkins. In Bandarban, the laboratory facility included a microscope and staining resources for the malaria smears, and a dissecting microscope for examining mosquitoes. In addition, the laboratory had facilities for processing, labelling and organizing the specimens collected in the field.

At the ICDDR,B laboratory in Dhaka, facilities were available to carry out PCR testing on filter paper blood spots using real time PCR, and to carry out PCR testing for mosquito species. Because of interest in both blood stage malaria as well as the presence of gametocytes, the molecular tests for malaria included a real time PCR multiplex DNA[6] as well as a specific test for RNA of the gametocyte using real time reverse transcriptase-PCR [7]. Molecular markers for drug resistance using real time PCR were also detected [8]. Additionally with mosquitoes, an ELISA procedure was established for detecting sporozoites from mosquitoes [9]. The real time PCR multiplex methods had earlier been established at Johns Hopkins University and were transferred to Dhaka by providing training at Johns Hopkins, equipment and reagents.

### Data management

All data from the field was entered onto pre-coded forms, which were designed for direct data entry using a scanner. Field workers were trained on using the forms so the information could be read easily using this system. The system used ABBYY FlexiCapture 8.0 software for electronic data capturing and processing. After entry, the data was exported to Microsoft Office Access 2007 database. The data files were maintained at the data center in Dhaka and copies of the data were also sent to Johns Hopkins. Images of the scanned data forms were maintained in digital format in case there was a need to inspect the primary data source. The data forms used are described on Table 2.

**Table 2:** Data Forms Used To Collect Information During Field Work

	Form/Filename	Form Description
**1**.	Demographic Surveillance -Initial	Includes household census data, pregnancies, and mosquito bed net use

**2**.	Demographic Surveillance - Follow-up	Includes any births, deaths, in-migration, out-migration, new pregnancies, and new bed nets acquired during the past 4 months

**3**.	Socio-Economic Status	Includes information on land holdings, household assets, sources of water and basic amenities, household income

**4**.	KAP Module1 - Malaria Prevention and Vector Control Practices	Includes questions to measure level of knowledge of malaria causation and prevention, current use of insecticide-treated bed nets and indoor residual spraying, and sources and costs of bed nets.

**5**.	KAP Module2 - Malaria Recognition, care seeking, and Treatment	Includes questions to measure symptoms recognized by participants, the process of care seeking when symptoms are recognized, and barriers encountered in receiving care and the affect it has on care seeking behaviour.

**6**.	KAP Module3 - Malaria in Pregnancy	Includes questions to measure the level of awareness of malaria in pregnancy, the proportion of pregnant women who sleep under treated nets, sources and types of treatment for preventing and treatment of malaria in pregnancy, and the proportion receiving treatment during pregnancy

**7**.	KAP Module 4 - Communication about malaria	Includes questions to determine the best communication mechanism for spreading malaria knowledge

**8**.	Active Surveillance Form	Includes questions on malaria symptoms in the last 48 hours, 2 weeks, treatment received, and amount spent on treatment

**9**.	Passive Surveillance Form	Includes questions on malaria symptoms, how malaria was confirmed, and treatment received

**10**.	Laboratory Form	Laboratory results for smear, RDT and spot on filter paper

**11**.	Entomological Collection Form	Includes questions on time of day; structural designation; type of catch; number of mosquitoes; composition of the household; number, age and gender of those sleeping under bed net; animal counts

## Results

The field area, consisting of two unions, had an area of 179 square kilometers located in southern Bangladesh, near the border with Myanmar. The maps of the two unions are shown in Figure 1 highlighting the defined clusters, which were used for the purpose of organizing the data collection. At the time of the initial census in 2009 (Kuhalong) and in 2010 (Rajbila), the total population of the field area was 20,563 persons including 16,319 tribal and 4,244 non-tribal people (primarily Bengali). The tribal people were overwhelmingly Buddhist while the non-tribal people were overwhelmingly Muslim. The age and sex distribution are shown in Figure 2.

**Figure 3:**
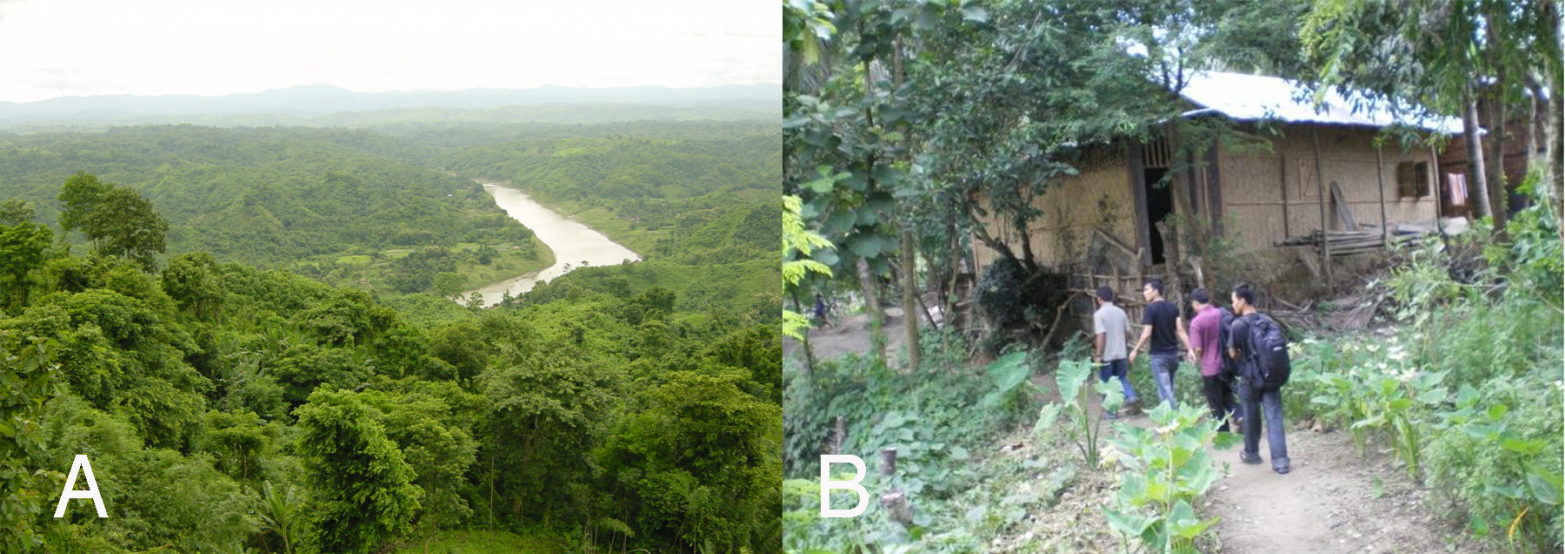
**Photos of hilly, forested terrain (A) and of surveillance workers (B)**.

The study area is hilly with elevations ranging from 80 to 152 meters. Vegetation consists of mixed thicket or dense forest with mixed evergreen and deciduous trees. The forested area is interspersed with some rice paddies. The average annual maximum temperature is 35.9°C; the average minimum is 13.2°C. The average total rainfall is 1780 mm, with most of the precipitation occurring from May through September. Photographs of the area are shown in Figure 3.

**Figure 4:**
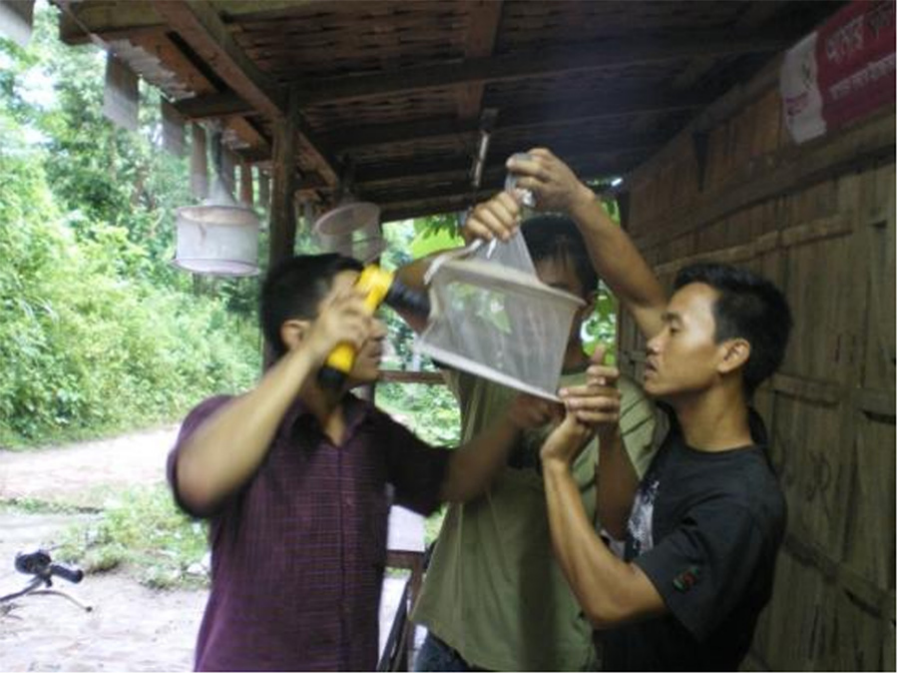
**Photo of team with the CDC light traps**.

*Anopheles *mosquitoes were found to be common throughout the year and a total of 22 different species of *Anopheles *were detected during the year. Eight of these species were found to be positive by CsELISA for sporozoites. Full details of these findings and of their relationship with the transmission of malaria in the area will be published elsewhere. Figure 4 shows the field team with the CDC light traps.

**Figure 5:**
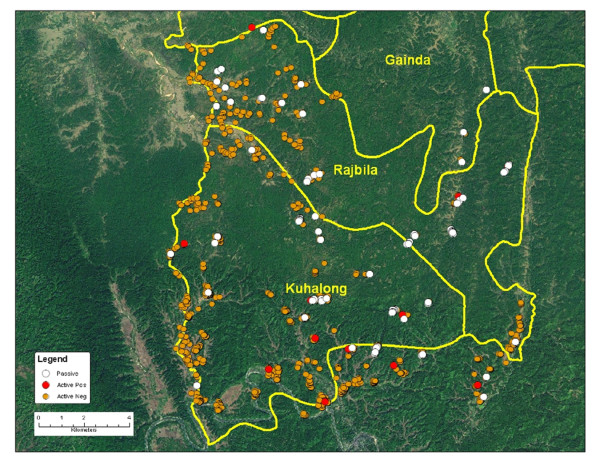
**Map showing the clustering of cases during the high season**. The orange dots show the locations of specimens from active surveillance which were negative. The other colored dots (red and white) show the locations of the positive specimens for active and passive surveillance, respectively. The union boundaries are political and not actual.

A total of 150 infections with malaria were detected from the field area by microscopy and RDT from the start of the surveillance through October 2010. Of these, 142 were *P. falciparum*, six were *P. vivax *and two were mixed infections. The distribution of the cases from active and passive surveillance in the two unions is shown in Table 3. Cases appeared to be highly clustered as shown in Figure 5. Although cases were detected throughout the year, most (84%) were found during the rainy season (May through September) when 80% of the total annual precipitation occurred. Cases appeared to be highly clustered as shown in Figure 5.

**Table 3:** Cases of malaria detected in the Bandarban field area through October 2010

	Active Surveillance	Passive Surveillance	Total
*Plasmodium falciparum*	21	121	142

*Plasmodium vivax*	1	5	6

Mixed	1	1	2

Totals	23	127	150

The malaria studies were found to be welcomed by the residents of the area and in fact 100% of the households agreed to participate in collection of the demographic, SES and KAP surveys, and 97% agreed to participate in the active surveillance which included blood draws. Furthermore, 100% of the households that were approached for mosquito trapping inside the house agreed.

## Discussion

The ICDDRB-JHU collaboration has established a population-based malaria research study area in the Hill Tracts region of Southern Bangladesh. This is an area which is close to the border with Myanmar and is considered hypoendemic for malaria. Unlike other areas with malaria in South Asian countries, this region is part of the same ecozone with Southeast Asia where malaria resistance has been described. Malaria is often considered to be primarily a problem of Africa and Southeast Asia, but the large number of cases observed highlights the importance of malaria in South Asia as well.

This paper describes the methodology used to establish this research field area; future reports will provide details of the specific studies being carried out.

During the first year of surveillance 150 cases of malaria were detected; mostly due to *P. falciparum*, though *P. vivax *and *Plasmodium malariae *were also detected by PCR. *Plasmodium ovale *has also been found in this area [10], but this study has not yet documented this species. There was marked seasonality with most cases being detected during the rainy warm season from May through August.

In contrast to limited number of *Anopheles *mosquito species found in Africa, the studies here have documented at least 22 such species in this area and eight of them were found to be positive for sporozoite antigen. In a previous study in Matiranga in the same eco-zone of Chittagong Hill Tracts 15 anopheline species were reported in the peak transmission season of 2009 of which seven were infected with sporozoite antigen [11]. With the great variety of species, there has been concern that bed nets may be less effective in this region because each species may have its own behavioural biting pattern. If the mosquitoes feed earlier in the evening, prior to bedtime, transmission may occur even though bed nets are being appropriately. Whether this concern is valid is not clear but requires additional study.

There has also been concern that the rapid test being used in the national programme detects only *P. falciparum*. This study will document the relative importance of the different species and this data will help to guide future interventions toward controlling malaria caused by all species of malaria.

In contrast to most malaria projects, this demographic surveillance system and malaria surveillance system includes all age groups, not only the groups who are thought to be at highest risk. Inclusion of all ages will facilitate epidemiological studies of age specific disease rates as well as studies of malaria transmission during the high and low seasons.

The inclusion of several ethnic groups who reside in the field area makes it possible for future studies to also identify specific risk factors for malaria among the different groups, depending on possible behavioural or genetic factors. A majority of the population belongs to Indian subcontinent tribal hill groups, which are known to have high rates of haemoglobinopathies [12] as well as different forms of housing construction. These factors may present risks for asymptomatic malaria transmission as well as symptomatic malaria infection. Additionally, many live in homes with raised bamboo flooring, allowing mosquitoes to enter the house more easily.

Combining the surveillance with a GIS and weather pattern database will allow for studies of geographic and climate risks. By studying patterns of malaria during the year, relationships between rainfall, temperature and other weather variable can be described. Differing rates of malaria from year to year may be the result of the interventions, or they may be more related to climate conditions which vary. These studies will help analyze these environmental factors.

This project includes molecular studies of malaria. PCR assays using filter paper blood spots increase the sensitivity of this surveillance for blood stage malaria and also allows for detection of infections due to non-falciparum malaria. RT-PCR is able to detect gametocytes from blood specimens; thus, detecting persons who transmit the infection. Identification of genes associated with resistance will allow for detection of resistant strains. PCR will also be used to confirm species identification of *Anopheline *mosquitoes.

## Conclusions

Looking ahead, this field area will be an important resource for the national programme to evaluate strategies for malaria control, as well as for research on the epidemiology of malaria, the transmission of malaria in a hypoendemic area, studies of genetic and behavioural risk factors, intervention studies to reduce transmission and for vaccine trials of promising new vaccines. The data can be shared with the Malaria Atlas Project [13]. In certain respects, this area of Bangladesh may be more suitable for vaccine trials since a moderate reduction in transmission may be observed more easily in this hypoendemic area with its established DSS and excellent active and passive surveillance, than in hyperendemic areas of Africa.

## Abbreviations

ICDDR,B: International Center for Diarrhoeal Disease Research, Bangladesh; JHMRI: Johns Hopkins Malaria Research Institute; UHFP: Upazila Health and Family Planning; RDT: Rapid Diagnostic Tests; ACT: Artemesinin based Combination Therapy; GPS:Global Positioning System; GIS: Geographic Information System;

## Competing interests

The authors declare that they have no competing interests.

## Authors' contributions

WK, DSa, RH, GG, MN, DN, SA, AC, DSu conceived and designed the study. ZH supervised GIS mapping with Assistance of SA, JK, MR, WK. WK, DSa, SA, CP, MSA, RH, JK, MR, JA, MMN, DN, GG, TS, ZH, DS analyzed the data. WK, DSa, SA, CP, MSA, RH, JK, MR, MMN, DN, GG, TS, ZH, AC, DS wrote the paper.
